# Thyroid hormone sensitivity indices and hs-CRP as biochemical predictors of arteriovenous fistula maturation in diabetic nephropathy

**DOI:** 10.5937/jomb0-61296

**Published:** 2026-03-17

**Authors:** Dan Li, Bing Zhao, Lihong Zou

**Affiliations:** 1 The Third Affiliated Clinical Hospital of Changchun University of Chinese Medicine, Department of Nephrology, Changchun, China

**Keywords:** thyroid hormone sensitivity, biochemical markers, hs-CRP, diabetic nephropathy, arteriovenous fistula maturation, osetljivost na tireoidne hormone, biohemijski markeri, hs-CRP, dijabetička nefropatija, sazrevanje arteriovenske fistule

## Abstract

**Background:**

Autogenous arteriovenous fistula (AVF) maturation is essential for successful hemodialysis in patients with diabetic nephropathy (DN). However, its outcome is often unpredictable. Thyroid hormone sensitivity indices and biochemical markers may provide valuable laboratory predictors for AVF maturation.

**Methods:**

A total of 102 DN patients undergoing AVF surgery were retrospectively analyzed and classified into mature and immature AVF groups. Laboratory data, including thyroid hormone sensitivity indices [free triiodothyronine (FT3), free thyroxine (FT4), thyroid-stimulating hormone (TSH), FT3/FT4 ratio, thyroid-stimulating hormone index (TSHI), thyroxine resistance index (TT4RI), thyroid feedback quantile index (TFQI), and parameterized TFQI (PFTQI)], as well as biochemical markers such as high-sensitivity C-reactive protein (hs-CRP), albumin, total cholesterol, triglycerides, and low-density lipoprotein cholesterol, were compared. Doppler ultrasonography was used as supportive assessment. Logistic regression identified independent predictors of AVF maturation.

**Results:**

Patients in the AVF Maturity Group exhibited significantly lower FT3, TSH, FT3/FT4 ratio, TSHI, TFQI, and PFTQI levels, along with reduced hs-CRP values (all P &lt; 0.05). Multivariate logistic regression revealed that higher FT4, TFQI, and PFTQI, lower hs-CRP, larger cephalic vein diameter, and younger age were independently associated with successful AVF maturation.

**Conclusions:**

Thyroid hormone sensitivity indices and hs-CRP serve as important biochemical predictors of AVF maturation in DN patients. These laboratory parameters may assist in risk stratification and clinical decision-making, providing a biochemical perspective for optimizing dialysis access outcomes.

## Abstract (sr)

**Uvod:**

Sazrevanje autogene arteriovenske fistule (AVF) je neophodno za uspešnu hemodijalizu kod pacijenata sa dijabetičkom nefropatijom (DN). Međutim, njen ishod je često nepredvidiv. Indeksi osetljivosti na hormone štitne žlezde i biohemijski markeri mogu pružiti vredne laboratorijske prediktore za sazrevanje AVF.

**Metode:**

Ukupno 102 pacijenta sa DN koji su podvrgnuti operaciji AVF retrospektivno su analizirani i klasifikovani u grupe zrele i nezrele AVF. Upoređeni su laboratorijski podaci, uključujući indekse osetljivosti na hormone štitne žlezde [slobodni trijodotironin (FT3), slobodni tiroksin (FT4), tireo-stimulirajući hormon (TSH), odnos FT3/FT4, indeks tireo-stimulirajućeg hormona (TSHI), indeks rezistencije na tiroksin (TT4RI), indeks kvantila povratne sprege štitne žlezde (TFQI) i parametrizovani TFQI (PFTQI)], kao i biohemijski markeri kao što su C-reaktivni protein visoke osetljivosti (hs-CRP), albumin, ukupni holesterol, trigliceridi i holesterol lipoproteina niske gustine. Doplerova ultrasonografija je korišćena kao pomoćna procena. Logistička regresija je identifikovala nezavisne prediktore sazrevanja AVF.

**Rezultati:**

Pacijenti u grupi zrelosti AVF pokazali su značajno niže nivoe FT3, TSH, odnosa FT3/FT4, TSHI, TFQI i PFTQI, zajedno sa smanjenim vrednostima hs-CRP (sve P &lt; 0,05). Multivarijantna logistička regresija je pokazala da su viši FT4, TFQI i PFTQI, niži hs-CRP, veći prečnik cefalične vene i mlađa starost nezavisno povezani sa uspešnim sazrevanjem AVF.

**Zaključak:**

Indeksi osetljivosti na hormone štitne žlezde i hs-CRP služe kao važni biohemijski prediktori sazrevanja AVF kod pacijenata sa DN. Ovi laboratorijski parametri mogu pomoći u stratifikaciji rizika i kliničkom donošenju odluka, pružajući biohemijsku perspektivu za optimizaciju ishoda pristupa dijalizi.

## Introduction

Diabetic nephropathy (DN) is a common and prevalent disease in the field of nephrology, with most patients progressing to end-stage kidney disease requiring maintenance hemodialysis (HD) treatment. Having a well-functioning and easily-maintainable dialysis access is crucial during the hemodialysis process [1]. Currently, the main types of dialysis access in clinical practice include synthetic grafts, venous catheters, and autogenous arteriovenous fistula (AVF) [2].

The maturation of AVF and the patient's recovery play a significant role in determining the timing and effectiveness of later-stage treatment for diabetic nephropathy. However, AVF also face early dysfunction issues, and the risk of early AVF dysfunction is much higher in DN patients undergoing hemodialysis compared to non-diabetic hemodialysis patients [3]. Thyroid hormone sensitivity indices are closely related to diabetic nephropathy, as thyroid hormones play a crucial role in the body's metabolism and hormone regulation, including their impact on glucose, fat, and protein metabolism [4].

For patients with diabetic nephropathy, ensuring the maturation of autologous AVF is the key to improve the quality of dialysis and life of patients. Mature autologous AVF can not only ensure efficient dialysis, reduce the complications such as infections and thrombosis, also can improve the patients survival rate and quality of life, reduce the consumption of medical resources for long-term and costs at the same time. Therefore, establishing and ensuring AVF maturation as early as possible is a crucial step in the dialysis treatment of patients with diabetic nephropathy. AVF is a surgical procedure that directly connects an artery to a vein and is often used in patients with chronic kidney disease who require long-term dialysis treatment [5]. AVF mature refers to the AVF after a period of growth and adaptation, attaining the state of a can be effectively used in hemodialysis [6]. This process usually takes weeks to months.

Thyroid hormone-sensitive hormones, such as Thyroid hormone sensitive indicators, such as free triiodothyronine (FT3), free thyroxine (FT4), Thyroid Stimulating Hormone (TSH), FT3/ FT4, Thyroid Stimulating Hormone index (TSHI), Index of thyrotropin resistance (TT4RI), Thyroid feedback quantile index (TFQI), The parameterized TFQI (PFTQI), etc. play important roles in metabolism, cardiovascular system, and overall health. In recent years, some studies have begun to focus on the thyroid hormone sensitive hormone function, the relationship between the state and AVF mature especially in dialysis patients with thyroid hormone sensitive hormones and the connection between the healthy blood vessels and the ability to repair [7].

The retrospective study was conducted to investigate the relationship between thyroid hormone sensitivity indicators and the maturity of AVF in 102 patients with DKD4-5 stage of diabetic nephropathy after AVF surgery, aiming to use laboratory indicators to predict the maturity of AVF in patients with diabetic nephropathy and improve the long-term prognosis of patients.

## Materials and methods

### Study design and participants

102 patients, who underwent AVF surgery, with DN stage 4-5 admitted to the Department of Nephro I ogy, the Third Affiliated Hospital of Changchun University of Traditional Chinese Medicine from June 2021 to August 2022, were included in this single-retrospective study. Inclusion criteria: (1) Patients who meet the aforementioned diagnostic criteria for DN and meet indications for hemodialysis; (2) Vascular assessment for autogenous arteriovenous fistula meeting the aforementioned criteria; (3) Age ≥ 35 years; (4) Surgical procedure involving forearm radial artery-to-cephalic vein autogenous fistula. Exclusion criteria: (1) Non-DN disease, type 1 diabetes, type 2 diabetes with autoimmune diseases, or malignant tumors; (2) Patients with a history of organ transplantation (e.g., kidney transplantation); (3) Patients with severe infections or coagulation disorders; (4) Pregnant or lactating women; (5) Patients with other organ failures.

This study was reviewed by the Medical Ethics Committee (Ethics Committee Approval Number: AF/SC-08/01.0). Informed consent was waived for this retrospective study due to the exclusive use of deidentified patient data, which posed no potential harm or impact on patient care.

### Grouping

According to the hospital's outcomes, the patients were divided into the AVF maturation group (n=72) and the AVF non-maturation group (n=30): those with AVF maturation and those without. The maturity of AVF should meet these criteria [8]: the vein diameter is ≥2.5 mm and the artery diameter is ≥2.0 mm under Doppler ultrasound; the blood flow rate is ≥500 mL/min. The vascular wall must be thick and elastic enough, without significant stenosis or thrombosis, and it should be possible to puncture it successfully to maintain stable blood flow velocity and pressure.

### Detecting methods

The diagnosis of stage 4-5 DN was established according to the Guidelines for the Prevention and Treatment of Diabetic Kidney Disease in China (2021 edition). Diagnostic confirmation required at least two of three consecutive measurements showing an elevated urinary albumin-to-creatinine ratio (UACR ≥30 mg/g) or urinary albumin excretion rate (UAE ≥30 mg/24 h or ≥20 μg/min) within 3-6 months, in conjunction with an estimated glomerular filtration rate (eGFR) <60 mL/min/1.73 m^2^ for more than three months, and/or characteristic renal biopsy findings. Stage 4 DN was defined as 15 ≤ eGFR < 29 mL/min/1.73 m^2^, while stage 5 was defined as eGFR < 15 mL/min/1.73 m^2^.

Venous blood samples were collected in the morning after overnight fasting. Samples were centrifuged at 3000 rpm for 5-10 min, and serum was promptly separated into sterile tubes. If immediate analysis was not feasible, specimens were stored at -20°C or below to ensure stability. Serum concentrations of FT3, FT4, and TSH were measured using standardized enzyme-linked immunosorbent assays (ELISA; Bell Biological Co., Beijing, China). High-sensitivity C-reactive protein (hs-CRP), albumin (ALB), serum calcium, phosphorus, total cholesterol (TC), triglycerides (TG), and low-density lipoprotein cholesterol (LDL-C) were determined by automated biochemical analyzers following the manufacturers' protocols and internal quality control procedures.

Vascular characteristics were evaluated using a color Doppler ultrasound system (Mindray DC-75, Shenzhen, China). Color Doppler was applied to assess vascular lumen morphology and flow direction, while pulse-wave Doppler was used to quantify blood flow velocity. Spectral Doppler analysis determined peak flow velocity and identified potential stenosis or vascular remodeling.

### Observation indicators

Baseline demographic and clinical data (age, sex, body mass index, duration of diabetes, primary etiology of chronic renal failure, and calcium channel blocker use) were collected. Laboratory biochemical indicators included hs-CRP ALB, serum calcium, phosphorus, TC, TG, and LDL-C. Thyroid-related laboratory indices (FT3, FT4, TSH) were used to calculate derived sensitivity indices: FT3/FT4 ratio, TSHI, TT4RI, TFQI, and PFTQI.

Complementary vascular ultrasound parameters were also obtained, including cephalic vein diameter, radial and brachial artery diameters, and corresponding blood flow velocities at baseline and two months post-AVF surgery.

### Statistical analysis

Data analysis was conducted with Statistic Package for Social Science (SPSS) 26.0 (IBM, Armonk, NY, USA). For continuous variables, normality tests were conducted, if the data follow normal distribution, continuous variables are presented as mean ± SD and compared using Student's t-test, while non-normally distributed variables are presented as median (inter-quartile range, IQR) and compared using Mann-Whitney U test. Categorical variables are expressed as numbers and percentages, and chi-squared test was used for comparison between groups. Risk factor analysis was conducted with multivariable logistic regression analysis. *P* <0.05 was considered statistically significant for differences.

## Results

### General information

A total of 102 subjects were included. The average age of the mature group was (60.3±10.22) years, and that of the immature group was (65.9±9.22) years. General clinical data analysis showed that there was no significant difference in age, male sex ratio, body mass index, and fasting blood glucose, glycated Hemoglobin A1c, albumin between the two groups (*P*>0.05). Use of calcium antagonists number comparative differences between groups with statistical significance (*P* < 0.05) (see Table 1 for details).

**Table 1:** Comparison analysis of general clinical data between two groups by Student's t test or chi-square test. Note: AVF: Autogenous arteriovenous fistula; HbAIC, Glycated Hemoglobin A1c.

Indicator	AVF Maturity Group<br>(72 cases)	AVF Immaturity Group<br>(30 cases)	t/χ^2^	P Value
Age (years, x̄±s)	60.3±10.22	63.9±9.22	1.667	0.099
Body Mass Index (x̄±s)	25.82±3.22	25.98±2.70	0.239	0.811
Male Cases (n, %)	42 58.33	19 63.33	3.819	0.052
Fasting Blood Glucose (mmol/L)	9.89±1.57	10.02±1.23	0.404	0.686
Calcium channel antagonists	58 80.55	20 66.66	4.711	0.029
HbAIC (%)	5.61±0.87	5.74±1.02	0.613	0.541
Albumin (g/L)	45.21±4.24	41.09±2.34	5.381	0.265
Duration of diabetes (years, x̄±s)	10.33±2.51	11.09±3.67	1.208	0.115

### Thyroid hormone & sensitivity indicators

The AVF Maturity Group generally shows lower levels of thyroid hormones (FT3, FT4) and related indices (TSH, FT3/FT4 ratio, TSHI, TFQI, PFTQI) compared to the AVF Immaturity Group (*P* < 0.05), (see Table 2 for details).

**Table 2:** Comparison analysis of thyroid hormone & sensitivity indicators between two groups by Student's t test or Mann-Whitney U test. Note: FT3: Free Triiodothyronine; FT4: Free Thyroxine; TSH: Thyroid Stimulating Hormone; FT3/FT4: Ratio of FT3 to FT4; TSHI: Thyroid Stimulating Hormone Index; TT4RI: Thyroid-Stimulating Hormone Resistance Index; TFQI: Thyroid Feedback Quantile Index; PFTQI: Parameterized TFQI.

Indicator	AVF Maturity Group<br>(72 cases)	AVF Immaturity Group<br>(30 cases)	* t/U *	* P value *
FT3 (pmol/L)	4.22±1.33	5.30±2.21	3.040	0.003
FT4 (pmol/L)	14.35±9.28	15.40±10.97	2.493	0.023
TSH (mlU/L)	2.1±0.34	2.69±1.52	3.130	0.002
FT3/FT4	0.36±0.01	0.38±0.01	9.203	<0.001
TSHI	3.20±1.30	4.33±0.98	4.276	<0.001
TT4RI	35.3±15.8	36.7±15.7	-1.083	0.027
TFQI	0.2 (-0.1, 0.4)	0.2 (0.0, 0.5)	-2.536	0.011
PFTQI	0.0 (-0.3, 0.2)	0.0 (-0.2, 0.3)	-2.542	0.011

### Comparison of biochemical indicators

Among these biochemical indicators, only hs-CRP shows a significant difference between the AVF Maturity and Immaturity Groups, with the Maturity Group having lower levels. The other indicators (ALB, TC, TG, LDL-C) do not exhibit statistically significant differences between the two groups (see Table 3 for details).

**Table 3:** Comparison of biochemical indicators by Student's t test (x̄±s). Note: hs-CRP: hypersensitive C-reactive protein; ALB: albumin; TC: Total cholesterol; TG: Triglyceride; LDL-C: Low density lipoprotein cholesterin

Group	hs-CRP (mg/L)	ALB (g/L)	TC (mmol/L)	TG (mmol/L)	LDL-C (mmol/L)
AVF Maturity<br>Group (n=72)	2.12±1.55	34.36±3.27	4.06±1.03	1.30±0.48	1.72±0.81
AVF Immaturity<br>Group (n=30)	4.10±2.99	34.17±5.56	3.82±0.75	1.21±0.38	2.10±0.51
* t *	2.552	0.163	1.153	0.913	0.750
* P value *	0.012	0.870	0.251	0.363	0.454

### Comparison of Doppler Ultrasonography

The Doppler Ultrasonography measurements reveal significant differences in vein and artery diameters between the AVF Maturity and Immaturity Groups, with larger diameters observed in the maturity group for both cephalic and radial arteries. Additionally, radial artery blood flow velocity is higher in the AVF Maturity Group compared to the Immaturity Group (see Table 4 for details).

**Table 4:** Comparison of Doppler ultrasound examination before and after treatment between the two groups by Student's t test (x̄±s). Note: ①Compared with the same group before treatment, P < 0.05; ②Compared with control group after treatment, P < 0.05.

Group	Cephalic Vein<br>Diameter (mm)		Brachial Artery<br>Diameter (mm)		Brachial Artery Blood <br>Flow (cm/s)		Radial Artery <br>Diameter (mm)		Radial Artery Blood <br>Flow (cm/s)	
	Before<br>treatment	After<br>treatment	Before<br>treatment	After<br>treatment	Before<br>treatment	After<br>treatment	Before<br>treatment	After<br>treatment	Before<br>treatment	After<br>treatment
AVF <br>Maturity	1.65± 0.22	2.42± 0.51①②	3.29± 0.52	4.09± 0.52	61.87± 12.45①②	70.32± 16.94	1.88±0.11	2.44± 0.62①②	47.99± 8.98	59.01± 15.11①②
AVF <br>Immaturity	1.54± 0.78	2.07± 0.40②	3.04± 0.97②	3.74± 0.88	59.98± 10.11②	67.12± 17.02	1.87± 0.12	2.10± 0.50②	48.18± 10.21	52.18± 13.04②
*t*	0.858	3.3505	1.437	2.4959	0.745	0.868	0.407	2.6621	0.093	2.1616
*P* value	0.393	0.0011	0.155	0.0142	0.458	0.3874	0.685	0.009	0.926	0.033

### Analysis of risk factors for maturity of internal fistula after AVF surgery

Higher FT3 levels (OR: 0.721, 95% CI: 0.567-0.915, *P* = 0.008) and a lower FT3/FT4 ratio (OR: 0.700, 95% CI: 0.457-0.802, *P* < 0.001) are associated with a lower likelihood of AVF maturity, while higher FT4 levels (OR: 1.111, 95% CI: 1.033-1.198, *P* = 0.004), higher TFQI (OR: 1.766, 95% CI: 1.162-2.684, *P*= 0.008), and higher PFTQI (OR: 1.654, 95% CI: 1.102-2.431, *P* = 0.012) are associated with a higher likelihood of AVF maturity. Additionally, older age (OR: 4.943, 95% CI: 0.776-0.984, *P* = 0.026), a larger inner diameter of the cephalic vein (OR: 5.023, 95% CI: 1.4-2.304, *P* = 0.025), and elevated hs-CRP levels (OR: 12.648, 95% CI: 0.171-0.600, *P* = 0.000) are also associated with a higher likelihood of AVF maturity (see Table 5 for details).

**Table 5:** Risk factors for maturity of internal fistula after AVF surgery were analyzed by multi-factor binary logistic regression analysis.

Covariate	OR	95%CI	P value	β	SE	Wald X^2^
FT3	0.721	0.567~0.915	0.008	-0.101	0.183	8.323
FT4	1.111	1.033~1.198	0.004	0.123	0.023	7.234
TSH	1.036	0.875~1.223	0.764	-1.137	1.082	1.134
FT3/FT4	0.700	0.457~0.802	<0.001	1.033	0.231	14.232
TSHI	1.266	0.942~1.235	0.098	-0.135	1.395	0.245
TT4RI	1.005	0.982~1.012	0.264	0.665	0.135	1.024
TFQI	1.766	1.162~2.684	0.008	-1.137	0.123	7.831
PFTQI	1.654	1.102~2.431	0.012	2.833	0.466	10.235
Age	4.943	0.776~0.984	0.026	-0.135	0.132	5.672
Calcium channel	0.286	0.170~2.245	0.592	0.665	0.134	1.011
hs-CRP	12.648	0.171~0.600	<0.001	-1.137	0.018	16.438
Inner diameter of<br>cephalic vein	5.023	1.4~2.304	0.025	2.002	0.774	6.583

## Discussion

Diabetic microvascular complications are one of the common complications in patients with type 2 diabetes. Good glycemic control can prevent the occurrence and progression of microvascular complications. The mechanism by which high blood sugar induces microvascular complications is complex and involves such factors as diabetes duration, dyslipidemia, age, body mass index, homocysteine, and thyroid dysfunction [9]. Controlling the progression of these complications is an important goal for improving clinical outcomes in patients. This study investigated the effect of thyroid hormone sensitivity indicators on the maturity of patients with diabetic nephropathy after AVF, which is a relatively novel line of research. The study found that thyroid hormone sensitivity indicators were significantly correlated with the risk of maturity of AVF. Specifically, FT3, FT4, FT3/FT4 ratio, TSHI, TT4RI, TFQI, PFTQI and other indicators in the mature AVF group were lower than those in the immature AVF group. In addition, age, hs-CRP and cephalic vein diameter are independent risk factors for AVF maturation. This association implies that clinical monitoring and regulation of thyroid hormone sensitivity indicators may improve the success rate of AVF and provide more effective means of dialysis for patients with diabetic nephropathy [10].

Research has found that in Type 2 Diabetes Mellitus patients [11] with normal thyroid function, low FT3 is an independent risk factor for DN, but is not associated with diabetic peripheral neuropathy. FT4 and TSH are not associated with any of these complications, which may be related to the metabolic processes of these two hormones in the body, with FT3 being the hormone that ultimately acts on various organs, rather than FT4 and TSH [12]. Thyroid hormone sensitivity indices are not exclusively associated with DN. It is generally acknowledged that chronic DN and end-stage kidney disease of any etiology can affect thyroid hormone parameters. However, a more meaningful exploration lies in the relationship between DN and thyroid hormones. In DN patients, pathological processes such as insulin resistance, glycolipid metabolic disorders, and renal injury may influence the synthesis, transport, and peripheral metabolism of thyroid hormones through multiple mechanisms. Conversely, thyroid hormone abnormalities may also participate in the occurrence and progression of DN, indicating a complex bidirectional interaction between them. Clarifying this association may provide new perspectives for the early diagnosis, condition assessment, and intervention of DN.

The relationship between the THS and AVF maturation mechanisms remains unclear. Current research focuses on thyroid hormones' cardiovascular impacts, but their specific roles and mechanisms in AVF maturation are unknown. AVF maturation involves endothelial cell proliferation, migration, and vascular remodeling. Thyroid hormones may influence AVF maturation by regulating endothelial function, collagen metabolism, and inflammation. However, existing studies lack in-depth exploration of the direct link between THS and AVF maturation. Also, the relationship between thyroid hormone levels and AVF maturation may vary among individuals due to different sensitivities. Future research should investigate the specific mechanisms between THS and AVF maturation to identify new therapeutic targets for improving AVF maturation. The elevated level of hs-CRP is associated with impaired maturation of AVF. As an inflammatory marker, hs-CRP signifies existing inflammation in the body. Inflammation can damage vascular endothelial cells, affecting vascular remodeling and maturation, thus impacting AVF maturation. Moreover, inflammation may hinder the proper maturation of AVF by influencing local hemodynamics and promoting thrombosis.

AVF creation involves surgically connecting a peripheral artery to a superficial vein, allowing arterial blood to flow into the superficial vein and arteriovenousization, thereby providing blood flow support for adequate hemodialysis. Currently, AVF creation is widely used in DN patients undergoing long-term hemodialysis treatment and other patients with chronic kidney disease. Generally, AVF maturation requires 4 to 8 weeks, and current researches on the mechanisms of poor AVF maturation mainly focus on two aspects: excessive intimal hyperplasia and dysfunctional outward remodeling [13]. Hernandez et al. [14] constructed a diabetic rat AVF model and found that vascular reactivity abnormalities in diabetic rats are mainly caused by excessive generation of superoxide anions, which are free radicals that play important roles in cell signaling, oxygen sensing, and inflammatory responses. Excessive generation of superoxide anions can cause tissue damage, and high blood glucose levels increase vascular oxidative stress and endothelial dysfunction, promote the release of pro-inflammatory cytokines, exacerbate the inflammatory response of the fistula vessels, prolong AVF maturation time, and neutralize endogenous endothelium-derived nitric oxide or other vasodilators, leading to vascular sclerosis, and further confirming the adverse effects of diabetes on AVF maturation. In addition, several studies have shown that cephalic vein diameter is an important factor in AVF maturation, but the underlying mechanisms are still not clear [15]. The findings of this study are generally consistent with the above-mentioned reports.

Thyroid hormone sensitivity index, as the response degree of target tissues to thyroid hormones, is used to evaluate the status of thyroid function. Thyroid hormones have a promoting effect on the proliferation and migration of endothelial cells, which is beneficial for the process of vascular reconstruction. Abnormal thyroid hormone sensitivity index may have adverse effects on the vascular maturation of AVF. In addition, abnormal thyroid hormone sensitivity index may affect the immune system and inflammatory response [16]. DN patients often have immune dysfunction and chronic inflammation, which are crucial for the successful maturation of the fistula. Abnormal thyroid hormone levels may lead to changes in immune system function, thereby affecting postoperative inflammatory response and immune regulation, which are unfavorable factors for fistula maturation. It is well known that thyroid hormones regulate basal metabolic rate and energy expenditure, which is crucial for maintaining and regulating nutritional status. Abnormal thyroid hormone sensitivity index may affect the nutritional status and physical recovery of DN patients, thereby affecting fistula maturation [17][18]. This study showed that FT3, TSH, FT3/FT4, TSHI, TT4RI, TFQI, PFTQI, and other indicators were lower in the mature AVF group compared to the immature group. Univariate logistic regression analysis also found that, except for TSH, TT4RI, and TSHI, the other variables were significantly associated with the risk of AVF maturation after surgery.

Furthermore, the results of the multivariable logistic regression analysis in this study showed that age, hs-CRP and cephalic vein diameter were independent risk factors for AVF maturation. This suggests that older age, inflammatory status, and smaller cephalic vein diameter pose greater challenges for establishing AVF in DN patients. The multivariable logistic regression analysis results showed that serum FT3 was not an independent risk factor for poor AVF maturation. This may be due to the small sample size in this study, interference of other factors on serum FT3 levels, and the fact that blood test results can only represent the body's state at the moment of blood sampling. Further evaluation of its correlation is needed through more dynamic monitoring and averaging of the results. In addition, the results of this study showed no significant correlation between gender, ALB levels, lipid levels, radial artery diameter, brachial artery diameter, brachial artery blood flow, and AVF maturation. This conclusion differs from previous research findings. Kordzadeh et al. [19] pointed out in their analysis of factors influencing AVF maturation in DN patients that hypoalbuminemia is an important factor leading to AVF immaturity, but there are also studies that did not suggest a correlation between serum ALB levels and AVF maturation [20]. In addition, some researchers believe that gender, obesity, radial artery diameter, brachial artery diameter, and brachial artery blood flow affect AVF maturation [21]. Patients with preoperative radial artery diameter ≥1.5 mm have a higher postoperative AVF maturation rate, and female patients have smaller blood vessels than males, so it is believed that the AVF maturation rate in females is lower than that in males. Furthermore, obesity accelerates atherosclerosis, inflammatory response, and intimal hyperplasia, increasing the rate of AVF immaturity [22][23]. The reason for the inconsistency of the conclusions in this study with the above research results may be related to the sample size, follow-up time, and regional population differences.

The study's findings offer actionable insights for clinicians managing AVF maturation in DN patients. Elevated thyroid hormone sensitivity indices (e.g., FT4, TFQI, PFTQI) and hs-CRP emerged as strong predictors of AVF failure, suggesting their utility in preoperative risk stratification. Physicians could integrate these biomarkers into preoperative assessments to identify high-risk patients who may benefit from alternative access modalities (e.g., grafts) or closer postoperative surveillance. For instance, patients with hs-CRP > 4 mg/L might require more frequent Doppler ultrasonography to detect early maturation delays, enabling timely interventions such as angioplasty. Additionally, thyroid axis optimization (e.g., addressing subclinical hyperthyroidism or thyroid resistance) and anti-inflammatory strategies (e.g., statins, glycemic control) could be prioritized in patients with unfavorable biomarker profiles. While this study does not establish definitive diagnostic thresholds, the identified associations support further validation of biomarker-driven protocols to personalize AVF management, ultimately improving hemodialysis success rates in DN populations.

It is important to acknowledge several limitations of this study. Firstly, it is a single-center retrospective study, which may limit the generalizability of the findings to other populations. Validation in diverse populations is necessary to accurately assess the stability of the identified risk factors. Secondly, despite the use of multivariate logistic regression to identify independent predictors of AVF maturation, there may still be unmeasured or residual confounding factors that were not accounted for. These could include lifestyle factors (e.g., smoking, alcohol consumption), medication use (e.g., immunosuppressants, anticoagulants), or other underlying health conditions that might influence both thyroid function and AVF outcomes. The study was based on a small sample size of diabetic nephropathy patients undergoing AVF surgery, highlighting the need for further multi-center studies to verify the association between risk factors and poor AVF maturation. A large, multi-center study would enhance the representativeness of the findings by including a broader DN population, encompassing patients with varying degrees of disease severity, comorbidities, and diverse ethnic backgrounds. Additionally, the enrolled cases involved clinical practices and complex surgical procedures, introducing numerous influencing factors during the perioperative period or post-operative care protocols over time. These factors should be considered when interpreting the results and may impact the generalizability of the findings.

## Conclusion

In conclusion, age, hs-CRP and cephalic vein diameter are independent risk factors for AVF maturation. Elevated levels of thyroid hormone sensitivity index are associated with poor AVF maturation. However, this study is a single-center study with limited cases and a relatively short follow-up time, which has certain limitations. It is necessary to conduct multicenter randomized controlled clinical trials to avoid differences in surgical skills among different centers and further explore the factors that affect AVF maturation, to provide a basis for clinical interventions.

## Dodatak

### Acknowledgements

The authors express their appreciation to staff in the Third Affiliated Clinical Hospital of Changchun University of Chinese Medicine, for their technical assistance.

### Author contributions

Dan Li: Writing - original draft, Data curation, Formal analysis; Bing Zhao: Writing - review and editing, Data curation, Formal analysis, Investigation; Lihong Zou: Writing - review and editing, Conceptualization, Formal analysis.

### Funding sources

This research did not receive any specific grant from funding agencies in the public, commercial, or not-for-profit sectors.

### Ethics and informed consent statement

This study was reviewed by the Medical Ethics Committee of the Third Affiliated Clinical Hospital of Changchun University of Chinese Medicine (Approval Number: AF/SC-08/01.0). Informed consent was waived for this retrospective study due to the exclusive use of de-identified patient data, which posed no potential harm or impact on patient care.

### Conflict of interest statement

All the authors declare that they have no conflict of interest in this work.
